# Male-specific Fruitless isoforms have different regulatory roles conferred by distinct zinc finger DNA binding domains

**DOI:** 10.1186/1471-2164-14-659

**Published:** 2013-09-27

**Authors:** Justin E Dalton, Justin M Fear, Simon Knott, Bruce S Baker, Lauren M McIntyre, Michelle N Arbeitman

**Affiliations:** 1 Biomedical Sciences Department and Program in Neuroscience, Florida State University, College of Medicine, Tallahassee, FL 32303, USA; 2 Genetics and Genomics Graduate Program, University of Florida, Gainesville, FL, USA; 3 Genetics Institute, University of Florida, Gainesville, FL, USA; 4 Department of Molecular Genetics and Microbiology, University of Florida, Gainesville, FL, USA; 5 Cold Spring Harbor Laboratory, One Bungtown Road, Cold Spring Harbor, NY 11724, USA; 6 Janelia Farm Research Campus, HHMI, 19700 Helix Drive, Ashburn, VA 20147, USA; 7 Department of Biomedical Sciences, Florida State University, 1115 West Call Street, Tallahassee, FL 32306, USA

**Keywords:** *Fruitless*, Sex hierarchy, Drosophila, Behavior, Genomics, RNA-seq

## Abstract

**Background:**

*Drosophila melanogaster* adult males perform an elaborate courtship ritual to entice females to mate. *fruitless* (*fru*), a gene that is one of the key regulators of male courtship behavior, encodes multiple male-specific isoforms (Fru^M^). These isoforms vary in their carboxy-terminal zinc finger domains, which are predicted to facilitate DNA binding.

**Results:**

By over-expressing individual Fru^M^ isoforms in *fru*-expressing neurons in either males or females and assaying the global transcriptional response by RNA-sequencing, we show that three Fru^M^ isoforms have different regulatory activities that depend on the sex of the fly. We identified several sets of genes regulated downstream of Fru^M^ isoforms, including many annotated with neuronal functions. By determining the binding sites of individual Fru^M^ isoforms using SELEX we demonstrate that the distinct zinc finger domain of each Fru^M^ isoforms confers different DNA binding specificities. A genome-wide search for these binding site sequences finds that the gene sets identified as induced by over-expression of Fru^M^ isoforms in males are enriched for genes that contain the binding sites. An analysis of the chromosomal distribution of genes downstream of Fru^M^ shows that those that are induced and repressed in males are highly enriched and depleted on the X chromosome, respectively.

**Conclusions:**

This study elucidates the different regulatory and DNA binding activities of three Fru^M^ isoforms on a genome-wide scale and identifies genes regulated by these isoforms. These results add to our understanding of sex chromosome biology and further support the hypothesis that in some cell-types genes with male-biased expression are enriched on the X chromosome.

## Background

**Figure 1:**
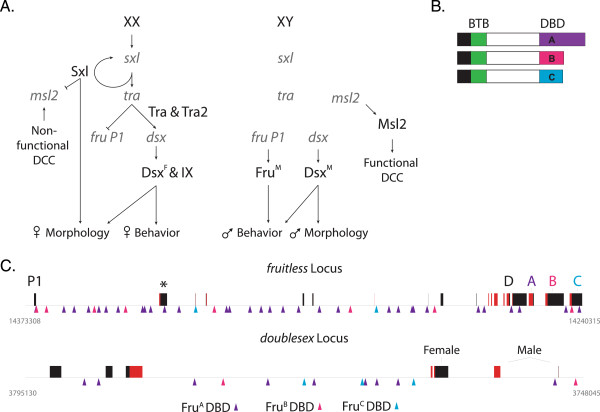
***Fruitless, *a gene in the sex-determination hierarchy, encodes multiple male-specific isoforms with distinct zinc finger domains. (A)** The Drosophila somatic sex determination hierarchy has two branches--one regulates dosage compensation and the other somatic sexual development. In females (chromosomally XX) Sxl is produced and so dosage compensation is not active. In males there is no Sxl and dosage compensation is active, resulting in increased expression of the single male X chromosome. For somatic sexual development, Sxl regulates the splicing of its own pre-mRNA and the *transformer* (*tra*). The product of *tra* (Tra) along with Transformer-2 (Tra2), coordinate to regulate splicing of transcripts produced from *doublesex* (*dsx*) and the P1 promoter of *fruitless* (*fru P1*). This branch of the hierarchy culminates in the production of sex-specific transcription factors (Dsx^F^, Dsx^M^, and Fru^M^) that specify sex-specific morphology and behaviors. Grey indicates transcripts; black indicates proteins. **(B)** Schematic of Fru^M^ proteins. Male-specific 101 amino acid region (Black), a bric-a-brac, tramtrack, broad-complex domain (BTB), and distinct zinc finger domains (A, B, or C) are indicated. **(C)***fruitless* and *doublesex* locus. Coding exons (red bars), non-coding exons (black bars), sex-specifically spliced exon of *fru* (asterisks), first *fru* promoter (P1), exons encoding the zinc-finger DNA binding domains (A-D), and male- and female-specific exons for *dsx* are indicated. The DNA binding motifs (triangles) for A (purple), B (pink) or C (cyan) DNA binding domains of Fru^M^ are indicated.

In *Drosophila melanogaster* differences in adult reproductive behaviors are specified by the somatic sex determination hierarchy (hereafter called sex hierarchy), a multi-branched hierarchy with functions in directing both sexual development and dosage compensation (Figure 1) reviewed in [1,2]. The branch of the sex hierarchy critical for specifying adult behaviors consists of a pre-mRNA splicing cascade that regulates the sex-specific splicing of transcripts from *doublesex* (*dsx*) and *fruitless (fru)* (Figure 1) reviewed in [3,4]. *fru* was initially shown to be important for male courtship behavior based on the phenotypes of mutant males that displayed high levels of male-male courtship behaviors [5]. This was distinct from the phenotypic observations with respect to other mutants that impacted courtship behaviors, in that the phenotype of the *fru* mutant was specific to courtship behaviors. Later, molecular-genetic analyses of *fru* demonstrated the position of *fru* in the sex hierarchy, and showed that it was required for all aspects of male courtship behaviors, providing strong evidence that *fru* is a key regulator of male courtship behavior [6-10].

*fru* is a complex locus that encodes both sex-specific and non-sex-specific proteins through the production of transcripts from at least four different promoters (*P1-4*) [6,7]. Transcripts expressed from the *fru P1* promoter are critical for male courtship behaviors, and are the only *fru* pre-mRNAs that are spliced by the sex hierarchy (Figure 1). *fru P1* transcripts produce multiple male-specific isoforms (Fru^M^) in ~ 2-5% of all central nervous system (CNS) neurons and these neurons have been shown to be important for courtship behaviors [11-14]. *fru P1* expressing neurons are present in both males and females [6,11,13,14], but the Fru^M^ protein isoforms are produced only in males where they contribute to building the potential for male courtship into the nervous system during development [15-18]. Conversely, *fru P1* transcripts in females are not translated [19,20]. All Fru isoforms are members of a family of conserved proteins that contain a BTB (BTB for *bric-a-brac, tramtrack, broad-complex*) domain and a zinc finger domain (Figure 1). Fru^M^ isoforms contain an amino-terminal 101 amino acid region of unknown function that is not present in Fru isoforms common to both sexes. *fru P1* transcripts are alternatively spliced at their 3′ ends into one of five exons that encode different zinc finger domains, which are predicted DNA binding domains (DBD; named A-E) [6,19,21,22]. Thus, *fru* is predicted to encode transcription factors. However, there is no direct evidence of Fru^M^ transcriptional activities, other than association with known chromatin modifying proteins [20].

Three of the five predicted Fru^M^ isoforms have been shown to be the predominate isoforms in adult head and central nervous system tissues (Fru^MA^, Fru^MB^ and Fru^MC^) [22]. These isoforms display differences in their expression patterns and in their ability to rescue male courtship defects [22]. As a first step to mechanistically understanding how Fru^M^ isoforms specify the potential for male courtship behaviors, the DNA binding specificities of each Fru^M^ isoform needs to be determined and the sets of genes that are regulated downstream of each Fru^M^ isoform identified. The identification of genes regulated by each Fru^M^ isoform will also contribute to our understanding of how *fru* functions to establish the potential for sex-specific behaviors.

Here, we identify genes that are induced or repressed by Fru^M^ by examining gene expression in adult head tissues where we over-express individual Fru^M^ isoforms (Fru^MA^, Fru^MB^ and Fru^MC^) in *fru P1*-expressing neurons of either males or females. We show that each isoform has different regulatory activities and that the sex of the fly impacts which genes change expression as a consequence of Fru^M^ over-expression. Similar over-expression conditions were previously used to demonstrate that Fru^M^ is sufficient, when expressed in female *fru P1*-expressing neurons, to specify the potential for nearly all aspects of male courtship behavior [4,12,14]. We used an *in vitro* binding site selection technique (SELEX) to identify the sequence motifs bound by each of three Fru^M^ isoforms and show that each isoform has different binding specificity [reviewed in [23,24]. For each gene, the coding sequence and the regulatory region (defined as 2 kb upstream and 2 kb downstream of the coding sequence) was examined for the presence of these binding sites. Genes containing these binding sites are enriched in the gene sets induced by over-expression of the respective Fru^M^ isoform in males, and in the genes identified as induced by Fru^M^ in loss-of-function mutant analyses. Additionally, genes induced by Fru^M^, are enriched on the X chromosome, whereas those that are repressed by Fru^M^ are under represented on the X chromosome.

## Results

The goal of our study was to identify genes whose expression was modulated (either up or down) by *fru P1* in the nervous system. To this end, we carried out a set of parallel experiments in which the GAL4/UAS system was used to overexpress each of the three best-characterized Fru^M^ isoforms (Fru^MA^, Fru^MB^ and Fru^MC^; Figure 1) [6,7,22], in just the *fru P1*-expressing neurons [14]. RNA-sequencing (RNA-seq) analysis was performed on mRNA extracted from the heads of such flies to identify differential expression on a genome-wide level, with exon-level resolution. We compared expression in heads in either male or female flies over-expressing one of the Fru^M^ isoforms, as compared to wild type adult male or female heads, respectively. This allows us to determine if there are differences in individual Fru^M^ isoform activities and if there are sex-specific factors that function in conjunction with Fru^M^. In addition, we examined gene expression differences in males mutant for *fru P1* (two different allele combinations; see Materials and Methods) compared to wild type males.

One of the strengths of RNA-seq analysis is the ability to detect differences in isoform expression levels. This is because exons are used for estimating expression, and thus the presence and difference in amount of transcript from alternative exon cassettes can be used directly to make inferences about isoforms. We focused on exon level expression and used existing models of transcript isoforms to identify alternative exon structures. Next, we tested for differential expression in our comparisons for each exon separately [see Materials and Methods and [25], and then used existing gene models to make inferences about differential expression of isoforms.

### Fru^M^ isoforms have different activities

To determine if Fru^MA^, Fru^MB^ and Fru^MC^ had different effects on gene expression in *fru P1*-expressing neurons we determined the gene sets that have exons that are both (1) statistically significantly induced or repressed and (2) have a ≥2 fold change in expression level when the data from animals overexpressing each Fru^M^ isoform are compared separately to the data from both CS and Berlin males (Additional file 1: Table S1). We found that over-expression of each Fru^M^ isoform leads to different subsets of genes with exons whose transcription is either induced or repressed. As expected, when we over-express each Fru^M^ isoform we found that the exon encoding the respective Fru DBD is significantly up regulated.

**Figure 2:**
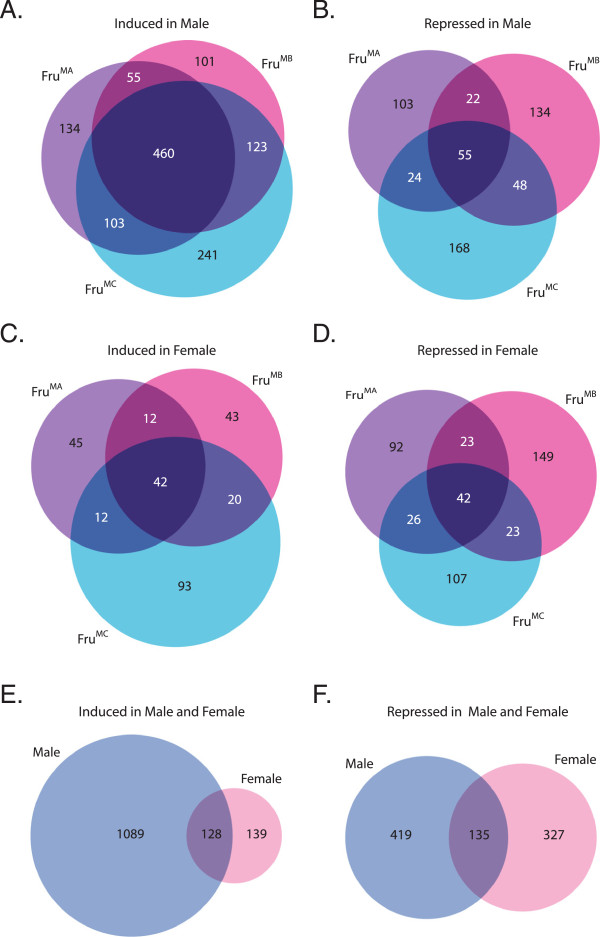
**Fru**^**M **^**isoforms have both distinct and overlapping sets of regulated genes. (A**-**F)** Venn diagrams displaying number of distinct and mutual genes between different sets of differentially expressed genes. **(A**-**D)** Numbers of genes induced or repressed in males or females by over-expression of Fru^MA^, Fru^MB^ or Fru^MC^ in *fru P1*-expressing neurons of the head. **(E**-**F)** Numbers of all genes induced or repressed by over-expression of any of the Fru^M^ isoforms.

Over-expression of Fru^MA^, Fru^MB^ and Fru^MC^ leads to 752, 739 and 927 genes with higher expression than wild type males, respectively. There were 460 genes with higher expression in all three conditions (Figure 2A and Additional file 1: Table S1; Additional file 2: Figure S1; Fru^M^-induced genes). We found that substantially more genes are up-regulated, than down regulated, relative to wild type expression. Over-expression of Fru^MA^, Fru^MB^ and Fru^MC^ lead to 204, 259 and 295 genes with lower expression than in wild type males, respectively. There are 55 genes with lower expression in all three conditions (Figure 2B and Additional file 1: Table S1; Fru^M^-repressed genes).

### Fru^M^ isoforms have different activities in males as compared to females

Using the same criteria as above, we examined the numbers of genes differentially expressed when Fru^M^ isoforms are over-expressed in female *fru P1*-expressing neurons as compared to wild type females of CS and Berlin strains. The rationale for these comparisons was to determine if there are additional sex-specific differences in *fru P1*-expressing neurons that might influence Fru^M^ activities. Over-expression of Fru^MA^, Fru^MB^ and Fru^MC^ in females leads to 111, 117 and 167 genes with higher expression than wild type females, respectively. There are 42 genes having higher expression in all three conditions, which included the exon encoding the respective Fru DBD (Figure 2C; Additional file 2: Figure S1 and Additional file 3: Table S2). Over-expression of Fru^MA^, Fru^MB^ or Fru^MC^ lead to 183, 237 and 198 genes with lower expression than wild type females, respectively. There are 42 genes with lower expression in all three conditions (Figure 2D).

These results further demonstrate that each Fru^M^ isoform has different activities with respect to regulating gene expression. In female *fru P1*-expressing neurons, more genes are repressed rather than induced when each isoform is expressed. This is in contrast to our observations in males. Taken together these findings suggest that the activity of each Fru^M^ isoform is influenced by the sex in which it is produced. This suggests that there may be other factors that are present in a sex-specific manner in *fru P1*-expressing neurons to influence Fru^M^ isoform activities.

Next, we determined if there are differences in the sets of genes that are induced or repressed by the over-expression of Fru^M^ isoforms in males and females. We determined the union of the genes that are induced by any of the three Fru^M^ isoforms in either males or females (Figure 2E and F). There are 1217 genes and 267 genes induced by any of the three Fru^M^ isoforms in males and females, respectively. The intersection of the induced genes in males and females is 128 genes (Figure 2E). We also determined the union of the genes that are repressed by any of the three Fru^M^ isoforms in either males or females. There are 554 genes and 462 genes repressed by one of the three Fru^M^ isoforms in males and females, respectively. The intersection of the repressed genes in males and females is 135 genes (Figure 2F). The genes induced in both males and females include *dsx*, *Gustatory receptor 93a*, *serotonin receptor 1A*, *semaphorin-5c* (Additional file 1: Table S1 and Additional file 3: Table S2). The genes repressed in males and females include *genderblind*, *methuselah-like 8* (Additional file 1: Table S1 and Additional file 3: Table S2). These genes are those for which over-expression of Fru^M^ isoforms in *fru P1*-expressing cells can influence gene expression independent of the sex of the fly.

Overall, many more genes are induced by overexpression of each Fru^M^ isoform in males as compared to females (Additional file 2: Figure S1), whereas there is not as large a difference in numbers of genes that are repressed by each isoform in males and females, suggesting that there is a sex-specific factor(s) that functions with Fru^M^ in males to facilitate gene induction.

### Fru isoforms have different DNA binding specificity

**Figure 3:**
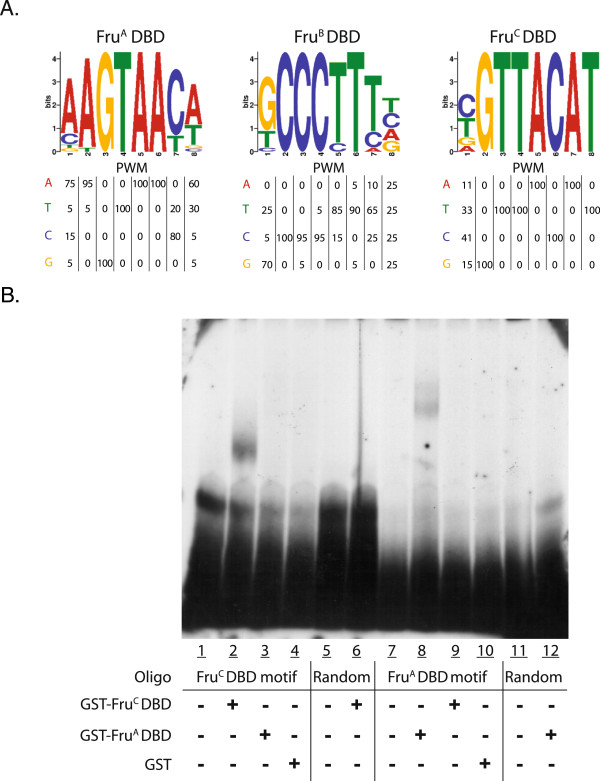
**Identification of DNA binding motifs for the A, B and C zinc finger domains of Fru**^**M**^**. (A)** Consensus DNA binding sequences of the zinc finger DNA binding domains (DBD) of Fru^MA^, Fru^MB^ or Fru^MC^ identified after ten rounds of SELEX. **(B)** Oligonucleotides containing the consensus sequences identified by SELEX, or sequences of the same nucleotide content but randomized in order, for DBD^A^ or DBD^C^ were incubated with GST-Fru^A^ DBD or GST-Fru^C^ DBD, or GST alone and assayed by gel mobility shift assay. GST-Fru^A^ DBD and GST-Fru^C^ DBD bound to oligonucleotides containing their respective consensus sequence (Lane 2 and 8) have slower mobility than GST (Lane 4 and 10), GST-Fru^A^ DBD (Lane 3 and 12), or GST-Fru^C^ DBD (Lane 6 and 9) incubated with oligonucleotides containing random sequences or the consensus sequence from other zinc finger domains or free oligonucleotides (no protein added in Lanes 1, 5, 7,11).

Our results demonstrate that Fru^M^ isoforms have different activities with respect to the gene sets that are induced or repressed in response to their expression. One possibility is that this is through differential DNA binding properties of the three isoforms. To address this question, we determined the binding site sequence for Fru^A^, Fru^B^ and Fru^C^ DBDs using an in vitro selection technique called SELEX (see Materials and Methods). From this analysis, we found that each of the DBDs examined bind different sequence motifs. The consensus motifs identified for the DBDs in the Fru^A^, Fru^B^ and Fru^C^ proteins are **AGTAAC**, **GCCCTTT**, and **TGTTACATCA**, respectively (Figure 3A).

Gel shift analysis, using purified GST-Fru DBD fusion proteins and the identified binding sites for Fru^A^ and Fru^C^, demonstrate that they each bind specifically to the sequence identified in the SELEX experiment, but do not bind to an oligonucleotide (oligo) containing a randomized sequence with the same nucleotide content (Figure 3B). Furthermore, Fru^A^ and Fru^C^ did not bind the oligos containing the motifs identified for Fru^C^ and Fru^A^, respectively (Figure 3B).

When gel shifts were performed using the DBD in the Fru^B^ protein, high levels of binding to DNA sequences that are present in each oligo used in these assays, flanking the SELEX identified motif, were observed in the gel shift assay (see Materials and Methods). This could be because the Fru^B^ protein has a poly-glutamine tract, which is not observed in the other binding domains and this facilitates non-specific association with DNA in the gel shift assay. To ensure that the correct sequence motif was identified for Fru^B^, the full SELEX technique was performed two independent times and identified nearly the same consensus sequence.

### Fru DNA binding site motifs are significantly enriched in genes that are induced by Fru^M^ isoforms

Next, we determined the frequency of each Fru binding site motif on a genome-wide level, in annotated gene regions, including introns and regions 2 kb upstream and downstream from each gene’s annotated start and stop site. We found that genes containing at least one sequence motif for Fru^A^ DBD are significantly enriched in the sets of genes that are induced downstream of Fru^MA^, and similar enrichments were observed for Fru^B^ and Fru^C^ in males, respectively (Table 1 and Additional file 4: Table S3). We do not see a similar enrichment when we examine the genes repressed downstream Fru^A^, Fru^B^ and Fru^C^ in males. A very different pattern is observed for genes induced downstream of Fru^A^, Fru^B^ and Fru^C^ in females, with only the gene sets induced downstream Fru^A^ showing enrichment of genes containing at least one Fru^A^ binding site (Table 1). These results suggest that the sets of genes that are induced in males by Fru^M^ over-expression includes genes that may be direct targets of Fru^M^, whereas those that are induced in females, or repressed in either males or females by Fru^M^ over-expression are not as likely to be direct targets.

**Table 1:** **Genes containing DNA binding motifs are significantly enriched in all Fru**^**M **^**-induced sets of genes in males**

			**Total Genes**	**Number of Genes with Motif**	**Expected Number of Genes**	**Chi-Square Value**	**Degrees of Freedom**	**Exact P-value for Chi-Square**	**Fisher raw P-value (2-tail)**
**Fru**^**A **^**DBD Motif**	Male	Induced by over-expression of Fru^MA^	14903	644	473.4123331	174.7423677	1	9.87E-39	2.39E-45
		Repressed by over-expression of Fru^MA^	14903	134	128.4256861	0.66217505	1	0.42340488	0.465483604
		Induced by Fru^M^ (As determined by *fru P1* mutant analysis)	14903	575	444.4536	108.65168	1	5.99946E-25	8.5546E-28
		Repressed by Fru^M^ (As determined by *fru P1* mutant analysis)	14903	276	274.4784272	0.023454623	1	0.880224856	0.919841345
	Female	Induced by over-expression of Fru^MA^	14903	80	69.87868215	3.98687096	1	0.04846709	0.04846709
		Repressed by over-expression of Fru^MA^	14903	116	115.2053949	0.014977928	1	0.9387153	0.938715296
**Fru**^**B **^**DBD Motif**	Male	Induced by over-expression of Fru^MB^	14903	378	183.7706502	287.4837937	1	3.99E-56	6.65E-56
		Repressed by over-expression of Fru^MB^	14903	50	64.40676374	4.36503163	1	0.041977653	0.035594233
		Induced by Fru^M^ (As determined by *fru P1* mutant analysis)	14903	309	175.564383	141.6955672	1	2.20046E-29	3.27561E-29
		Repressed by Fru^M^ (As determined by *fru P1* mutant analysis)	14903	128	108.4221969	4.847043913	1	0.028457111	0.032441531
	Female	Induced by over-expression of Fru^MB^	14903	35	29.09494733	1.607773993	1	0.236941316	0.199286144
		Repressed by over-expression of Fru^MB^	14903	64	58.93591894	0.588511944	1	0.448926212	0.44892621
**Fru**^**C **^**DBD Motif**	Male	Induced by over-expression of Fru^MC^	14903	296	131.8687513	253.964127	1	3.55E-46	4.53E-46
		Repressed by over-expression of Fru^MC^	14903	33	28.16614105	0.261120798	1	0.613295999	0.613295997
		Induced by Fru^M^ (As determined by *fru P1* mutant analysis)	14903	209	100.4307858	143.6364162	1	2.29794E-27	2.78422E-27
		Repressed by Fru^M^ (As determined by *fru P1* mutant analysis)	14903	62	62.0224116	9.72597E-06	1	1	1
	Female	Induced by over-expression of Fru^MC^	14903	30	23.75629068	1.934825144	1	0.180129952	0.180129951
		Repressed by over-expression of Fru^MC^	14903	33	28.08642555	0.980189811	1	0.355875025	0.306776226

Fru^A^, Fru^B^ and Fru^C^ sites are found in overlapping sets of genes and the presence of a motif for one significantly increases the likelihood of finding a binding site for at least one of the other. This result may explain why there were several genes that were induced by each of the three Fru isoforms in both males and females (see above).

*fru* has many binding sites with 32, 8 and 2 binding sites motifs for each Fru^A^, Fru^B^ and Fru^C^ DBD, respectively (Figure 1C). Additionally, *dsx* has a large number of binding sites with 9, 2 and 3 binding sites motifs for Fru^A^, Fru^B^ and Fru^C^ DBD, respectively (Figure 1C), although it should be noted that both *fru* and *dsx* are large genes. Fru^M^ may regulate *dsx* expression directly, consistent with the overlap observed between Dsx and Fru^M^ in the CNS [26,27], it may also regulate its own expression. This regulation of both *dsx* and *fru* transcript levels by Fru^M^ isoforms could ensure sufficient *dsx* and *fru* expression in neurons important for male courtship behaviors.

### Genes with roles in neuronal patterning and physiology are enriched among genes regulated by Fru^M^ isoforms in males and not in females

*fru P1*-expressing neurons are present in very similar positions and numbers in adult males and females. To gain insight into the processes Fru^M^ regulates, we examined gene ontology (GO) enrichment of protein domains [28] and biological processes, molecular functions and cellular processes [29] for Fru^M^ regulated genes (Additional file 5: Table S4). For the 1,217 genes up-regulated by over-expression of any of the Fru^M^ isoforms in males, there is an enrichment of genes that contain protein domains that function in neuronal patterning and physiology. Among the enriched categories for protein domains are immunoglobulin-like fold, pleckstrin homology domain, PDZ domain, Ion transport domain, epidermal growth factor-like domain, and voltage dependent potassium channel. Many proteins with these domains function at the plasma membrane and mediate neuronal projection patterns, form complexes with channels, and make junctions, including synaptic and neuromuscular junctions, which are consistent with functions ascribed to Fru^M^ isoforms (Additional file 5: Table S4).

Among the enriched GO terms for those 1,217 up-regulated genes are those that underlie diverse functions in development of the nervous system and adult physiological functions. The Biological Process GO terms include axon guidance, regulation of response to stimulus, regulation of neuron differentiation and behavior, among many others. The Cellular Component GO terms include synapse, ion channel and neuromuscular junction, among many others (for a complete list see Additional file 5: Table S4).

In contrast, an analysis of the 554 genes down-regulated by over-expression of any of the Fru^M^ isoforms in males revealed an enrichment of genes that contain protein domains that function in lipid and triglyceride metabolism, consistent with previous studies [30-32] (Additional file 5: Table S4).

An analysis of the genes induced by overexpression of Fru^M^ in females identified fewer significantly enriched GO Biological Process categories, as compared to our observations in males. These include response to caffeine, response to purines, and potassium ion transport. The GO category male sex differentiation is included, but only contains *dsx* and *fru*. Nearly all the enriched GO Biological Process categories identified in the genes repressed by Fru^M^ in females include defense response genes (Additional file 5: Table S4).

Genes that are up regulated in response to over-expression of Fru^M^ isoforms include those that were previously implicated in playing a role in *fru P1* expressing neurons, including the ecdysone receptor gene *EcR* and the ecdysone hierarchy gene *broad*[33]. Several neuronally-expressed genes, not previously known to be regulated by Fru^M^ isoforms, were identified that play critical roles in axon target recognition and attraction, axon guidance, dendrite guidance axon defasiculation, and sensory perception. These genes include *roundabout* (1 and 3), *Dscam* (1,2,3 and 4), *prospero*, *semaphorin* (1a, 2a), *Netrin A and B*, *fasiclin* (I and II), *Notch*, *Cadherin N*, *Gustatory receptor 93a* and *abnormal chemosensory jump 6, Gaba receptor, serotonin receptor,* nicotinic *Acetylcholine Receptor alpha 7E*, *Neuroligin 1*, *foxo*, *Target of rapamycin*, *cacophony*, *muscarinic Acetylcholine Receptor 60C*, *spinster*, and *Dopamine receptor 2,* among many genes.

An analysis of the genes induced in response to the expression of Fru^M^ isoforms reveals that a large fraction of these genes had previously been shown to have high expression in nervous system tissues. Thus of the genes with induced expression in response to Fru^M^ isoform expression, 649, 645 and 592 genes were previously shown to be significantly highly expressed in the adult brain, larval brain and adult ventral nerve cord, respectively, when compared to Flyatlas data using the Flymine portal [28,34]. Of the tissues examined in the Flyatlas study, these three tissues had the largest overlap of genes with significantly high expression with the genes induced by Fru^M^ from this study.

### Comparison of differential gene expression in response to Fru^M^ over-expression vs. Fru^M^ loss of function

We have also analyzed gene expression differences in head tissues of *fru P1* mutant males as compared to wild type males to further confirm our over-expression analysis. Over-expression of Fru^M^ is likely to yield higher fold-differences in gene expression than observed in the loss-of-function mutants, because the absolute difference in *fru P1* mRNA amounts is greater between over-expressor flies and wild type flies than between loss-of-function *fru P1* flies and wild type flies based on RNA-seq data. Thus, we identified genes based on significant differences in expression between *fru P1* mutants and wild type males, but did not require a ≥2 fold change.

Based on the loss-of-function analyses, of the 706 genes that are induced by Fru^M^, 209 genes were also induced by at least one of the Fru^M^ isoforms in the over-expression experiments. If we do not restrict the list of genes to those with ≥ 2-fold induction by overexpression of at least one of the Fru^M^ isoforms, 360 genes were identified as induced by Fru^M^ in both the loss-of-function and overexpression experiment. Of the 436 genes that are repressed by Fru^M^, 19 genes were also repressed by at least one of the Fru^M^ isoforms in the overexpression experiments (Additional file 2: Figure S1 and Additional file 6: Table S5). There is a significant association between the lists of genes that we identified as regulated downstream of *fru P1* in the loss-of-function analyses to those identified in the overexpression analyses (*p* <0.05).

The genes induced by *fru P1* in the *fru P1* loss-of-function analysis have a significant enrichment of genes that contained either the Fru^A^, Fru^B^ or Fru^C^ binding sites motif, whereas those that are repressed downstream of *fru P1* show an enrichment of only the Fru^B^ binding site motif (Table 1). Taken together, these results further support the idea that genes that are induced downstream of Fru^M^ isoform are likely direct targets.

### Genes that are regulated downstream of Fru^M^ isoforms do not have the expected chromosome distribution

**Figure 4:**
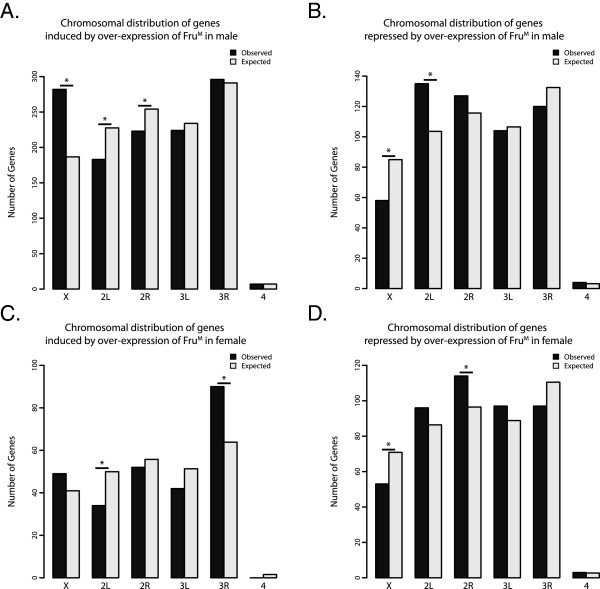
**Chromosomal distributions of genes regulated by Fru**^**M**^**. (A**-**D)** Observed (black) and expected (grey) number of genes on each chromosome for the sets of genes induced and repressed by over-expression of Fru^MA^, Fru^MB^, and Fru^MC^ in males or females. Asterisks indicate significant enrichment or depletion between observed and expected (Fisher’s exact test, p-value < 0.05).

An examination of the chromosomal distribution of the genes with exons that are either induced or repressed downstream of Fru^M^ isoforms in the male over-expression experiments revealed a significant enrichment and depletion on the X chromosome, respectively. The genes with up-regulated expression downstream of Fru^M^ isoforms in males are enriched on the X chromosome and the second chromosome (Figure 4 and Additional file 7: Table S6). The genes with reduced expression downstream of Fru^M^ isoforms are significantly depleted from the X chromosome. In addition, if we examine the genes that are induced by Fru^M^ that were previously shown to be significantly highly expressed in the adult brain, larval brain or adult ventral nerve cord these genes sets are present on the X chromosome at a higher level than expected, based on Flyatlas data using the Flymine portal [28,34]. These results suggest that the X chromosome has properties distinct from the autosomes with respect to genes important for the potential for male courtship behaviors.

## Discussion

In this study we identified hundreds of genes regulated downstream of Fru^M^ isoform activity. Fru^MA^, Fru^MB^ and Fru^MC^ have differences in the gene sets induced or repressed when they are over-expressed, demonstrating that each isoform has distinct biochemical activities (Figure 2). Consistent with this observation is that each Fru^M^ isoform has different DNA binding specificity (Figure 3). Our results suggest that there are sex-specific factors that influence Fru^M^ isoform activity, as over-expression of Fru^MA^, Fru^MB^ and Fru^MC^ isoforms in males and females resulted in different genes that are induced and repressed by each isoform (Figure 2 and Additional file 2: Figure S1). The gene sets identified as induced downstream of Fru^M^ isoforms in males are enriched with genes with nervous system function, based on GO annotations (Additional file 5: Table S4).

Additionally, it is worth noting that there may be other possible sources for the differences observed in the gene expression levels in these experiments. First, the Fru^M^ proteins contain a BTB domain that in previous work has been shown to contain a dimerization domain that can mediate homodimeric or heterodimeric interactions. Thus, some of the effects we observe could be due to 1) differences in the stoichiometric ratios of Fru^M^ with each other, and/or 2) differences in the stoichiometric ratios of Fru^M^ with other potential dimerization partners. However, based on immunofluorescence results we do not observe substantially different levels of over-expression of each isoform, nor is there substantial expression, if any, outside of the normal *fru P1* expression pattern (see Additional file 8: Figure S2). Second, there is a significant association between the genes that are either induced or repressed when Fru^M^ is over-expressed, with those genes identified in loss-of-function *fru*^*M*^ mutant analyses, demonstrating the physiological relevance of the genes identified by over-expression. Third, there is significant enrichment of the binding site sequences identified for each isoform within the genes that are induced by each isoform. Fourth, while our criteria were stringent (significant and substantial differences from two different wild type strains), strain differences may account for some of the differences between wild type male and female and Fru^M^ over-expressor male and female strains, respectively. However, such strain differences are not likely to account for the differences we observe between the Fru^M^ isoforms, which are in the same genetic background, nor can strain differences account for differences observed across sex. Taken together, these results demonstrate that in context of the over-expression experiments each of the three Fru^M^ isoforms examined has different activities with respect to genes that are induced or repressed in males, with many more genes having induced rather than repressed expression in males.

In previous studies, production of Fru^M^ in females, by expression of a *tra-2* RNAi transgene in *fru P1*-expressing neurons, was sufficient to endow females with the potential to perform the first four sub-steps of the male courtship ritual, following, tapping, wing extension and proboscis extension, but not attempted copulation [14]. In contrast, overexpression of Fru^MA^ or Fru^MC^ in *fru P1*-expressing neurons, resulted in flies that displayed only following and tapping reviewed in [4], suggesting that overexpression of Fru^M^ in females is not sufficient to endow females with the potential to perform courtship behaviors. The 42 genes identified as induced by all three Fru^M^ isoforms in females will be interesting to examine, with respect to their role in establishing the potential for these early courtship steps. Interestingly, one of these genes is *Ir54a*, which encodes a member of a diverse family of ionotropic receptors, some of which are expressed in the adult antenna and underlie chemosensory functions [35]. It is also known that Dsx^M^ plays a role in establishing the potential for courtship behaviors [26,27,36-38], which would not have been present in females in which Fru^M^ was produced, though Dsx^M^ is not present in all *fru P1*-expressing neruons, so is unlikely to account for all the differences between males and females observed here [26,37]. Our results may further explain why there was not a complete rescue of male courtship behavior. It is clear that the sex of the fly in which Fru^M^ is produced has an impact on the genes that are induced and repressed. These results suggest that there are additional sex-specific factors that influence Fru^M^ activity, which may include Dsx^M^. Further biochemical characterization of Fru^M^ protein interactions will be important to understand Fru^M^ activities.

While *fru* has been predicted to be a transcription factor based on the observation that *fru* encodes BTB-zinc finger products, no direct transcriptional targets of *fru* have been identified, leaving this an open question. A recent study has shown that Fru^M^ associates with a cofactor, Bonus, and subsequently associates with two chromatin modifying proteins, HP1a and HDAC1, however it was not clear if the association of Fru^M^ with chromatin was direct [20]. The results presented here demonstrate that Fru^M^ can bind DNA and that three Fru^M^ isoforms examined have different binding activities. Given our observation that the binding sites are significantly enriched in all gene sets identified as induced, but not repressed by Fru^M^, suggests that Fru^M^ may function by binding enhancer DNA directly, but acts in an indirect manner to repress gene expression (Table 1).

In previous studies we and others have show that genes with male-biased expression were enriched on the X chromosome in the adult head [31,39] and brain [40]. There was also a significant enrichment of genes with male-biased expression that reside near dosage compensation entry sites [39,40]. Here, we observe significant enrichment of genes that reside on the X chromosome that are induced by Fru^M^ in males. This observation supports the idea that over evolutionary time there may have been a selection for genes with male-specific functions to reside on the X chromosome and in particular those regulated by Fru^M^. Perhaps, Fru^M^ isoforms and their gene targets have evolved to take advantage of the unique properties of the male nucleus. These differences include the dosage compensation complex that is bound to the male X chromosome that leads to less compact chromatin reviewed in [41], the presence of the Y chromosome that affects chromatin architecture throughout the nucleus [42], or other differences in the chromatin and three-dimensional architecture of the nucleus in males [for example see [43]. It is possible that there are more interconnections in the sex hierarchy model between chromosomal sex, the sex hierarchy branches and sexual development that is downstream of Fru^M^ than shown in the model (Figure 1).

## Conclusions

The results in this study add to the information regarding Fru^M^ function with the identification of hundreds of genes regulated by Fru^M^, many of which have known roles in nervous system development and physiology. One of the next exciting challenges to our understanding of how complex behaviors are specified at a molecular-genetic level will be to develop tools to interrogate the functions of specific transcript isoforms in a cell-specific manner.

## Methods

### Flies

Flies were raised on standard cornmeal food medium at 25°C on a 12 hour light and 12 hour dark cycle. Wild type flies were the Canton-S (CS) and Berlin strains. Transheterozygous mutants for *fru P1* are *Df(3R)P14/Df(3R)fru*^*4-40*^ and *fru*^*w12*^*/Df(3R)Cha*^*M5*^, which are phenotypically void of all male courtship behaviors [10]. In addition, no detectable *fru P1* transcript or Fru^M^ protein is present in *Df(3R)P14/Df(3R)fru*^*4-40*^[11], and no full length *fru P1* mRNAs are present in *fru*^*w12*^*/Df(3R)Cha*^*M5*^[10]*. Df(3R)P14* contains a breakpoint in *fru* extending proximally removing common *fru* coding exons and ending in the cytological location 90C2-D1 [5]; *Df(3R)fru*^*4-40*^ contains a breakpoint in *fru* extends distally thus removing the *fru P1* promoter [11]; *fru*^*w12*^ is an inversion-cum-translocation that removes *P1-3* from common *fru* coding exons [10]; and *Df(3R)Cha*^*M5*^ contains a breakpoint between *P1* and *P2* that extends distally removing *P1* ending in the cytological location 91D [5,10]. The *fru P1-Gal4*, *UAS-Fru*^*MA*^*, UAS-Fru*^*MB*^, and *UAS-Fru*^*MC*^ were described previously [14,44]. *y*^*1*^*w*^*1118*^*; P(UAS-Gal4.H)12B* stock was obtained from Bloomington stock center.

Flies that ectopically expressed Fru^M^ isoforms were of the genotypes *y w/(w or* Y)*; P(w*^*+mC*^*, UAS-Gal4)/P(w*^*+mC*^*, UAS-Fru*^*MA*^*,*^*B*^*, or*^*C*^*); fru P1-Gal4/+*. While each UAS-Fru^M^ transgene is inserted at a different location, they are homozygous viable and each respective DNA binding domain (A, B, and C) encoding exon is > four-fold induced compared to wild type in our over-expression assay conditions, by examining the RNA-seq expression data. Immunofluorescence using an antibody specific to the male-specific region common to all Fru^M^ proteins demonstrates relatively similar levels of the male-specific proteins in *fru P1-Gal4* expressing neurons and is not readily detectable in other regions of the CNS (Additional file 8: Figure S2). Additionally, examination of male-female courtship of the above transgenic strains demonstrated that over-expression is not changing male behaviors significantly (Additional file 9: Figure S3).

### Tissue collection

Adult head cDNA libraries were prepared from three independent biological replicates from each of the following genotypes: 1) from males and females: Canton S, Berlin, *y w/(w or* Y)*; P(w*^*+mC*^*, UAS-Gal4)/P(w*^*+mC*^*, UAS-Fru*^*MA*^*); fru P1-Gal4/+*, *y w/(w or* Y)*; P(w*^*+mC*^*, UAS-Gal4)/P(w*^*+mC*^*, UAS-Fru*^*MB*^*); fru P1-Gal4/+*, *y w/(w or* Y)*; P(w*^*+mC*^*, UAS-Gal4)/P(w*^*+mC*^*, UAS-Fru*^*MC*^*); fru P1-Gal4/+* and 2) in males only*: Df(3R)P14*/*Df(3R)fru*^*4-40*^ and*, fru*^*w12*^*/Df(3R)Cha*^*M5*^ males. For each experimental condition, approximately 200 flies that were 8 to 24 hours post-eclosion were used. All flies were collected 0 to 16 hours post-eclosion under anesthetization and allowed to recover for 8 hours before being snap frozen in liquid nitrogen. Snap frozen whole animals were stored at -80°C until heads were collected. Adult heads were separated from bodies by mechanical tapping of the cryovial. A piece of plastic was cooled on dry ice, on which the frozen heads were separated from the bodies and immediately transferred and homogenized in 1 mL of TRIzol® (Invitrogen).

### Illumina sequencing library preparation

Total RNA was extracted using TRIzol® Reagent (Invitrogen), and RNA was precipitated by addition of 250 μL 100% isopropanol and 250 μL 1.2 M NaCitrate, 0.8 M NaCl in DEPC-treated H_2_O. Approximately 25 μg total RNA was DNase treated to remove any trace amounts of DNA, following Zymo Research RNA Clean & Concentrator™-25 In-Column DNase Digestion protocol, using 10 units Ambion® TURBO™ DNase. Poly(A) + transcripts were subsequently isolated from total RNA using Ambion® MicroPoly(A)Purist™ Kit. 100 ng mRNA was chemically fragmented to a range of approximately 200-500 base pairs using the Ambion® RNA Fragmentation Reagent, and the reaction was cleaned using Zymo Research RNA Clean & Concentrator™-5. First strand cDNA was synthesized using SuperScript® II Reverse Transcriptase (Invitrogen™) and a combination of 3 μg random hexamers and 0.15 μg oligo(dT)_20_ primers. Following first strand synthesis, the second strand of the cDNA was synthesized by addition of DNA polymerase I (Invitrogen™), RNase H (New England Biolabs® Inc.), dNTPs and second strand buffer (Invitrogen™). This reaction and all subsequent reactions were cleaned using Zymo Research DNA Clean & Concentrator™-5 kit. Double stranded cDNA templates were blunt ended using End-It™ Repair Kit (Epicentre®). Next, A-overhangs were then added to both ends with Klenow fragment (3′ → 5′ exo-minus) (New England Biolabs® Inc.). Illumina sequencing adapters were then ligated to both ends of the cDNA templates using Fast-Link™ DNA Ligation Kit (Epicentre®). cDNA templates were then amplified by performing polymerase chain reaction (PCR; 18 cycles) that extended the adapter and incorporated a different six base pair index into each sample. The product was then isolated by gel purification of 250-550 base pair fragments. Samples were then pooled and sequenced on the Illumina Genome Analyzer GAII platform with 72 base pair single end reads, and the reads were matched to their corresponding sample via the index.

### SELEX

Each of the three *fru* DNA binding domain (DBD) encoding sequences were PCR amplified from *fru* cDNAs using the following primer pairs that contain *EcoR*1 or *Xho*I restriction sites engineered at their ends: Fru^MA^ primers are 5′CCGGAATTCCGC GTCAAGTGTTTTAACATTAAGC and 5′CCGCTCGAGGTTTGCTTGATTCTTGGTTACTTA; Fru^MB^ primers are 5′GGC CGGAATTCTCCAAGGCCTGGCACATG and 5′ CCCGCTCGAGTGTGCTG CTGTTGCTGC; Fru^MC^ primers are 5′CCGGAATTCCAGCAGCGCCCGCCACC and 5′GCCGCTCGAGCGGGATGGGCTGCACTTGGGC. For each DBD-encoding exon, the first primer has the *EcoR*1 site and the second primer has the *Xho*I site. The primers were designed to amplify beginning where the divergent sequence for each Fru isoform begins (see Figure 1), and the amplicon includes each isoforms respective stop codon at the end. Each region was cloned as in-frame fusions with Glutathione S-transferase at the amino terminus (GST-Fru), into the pGEX-4 T1 plasmid for expression in bacteria.

Each DBD containing plasmid was transformed into *E.coli* BL21 and single colonies were grown to ~ OD_600_ = 0.8 when IPTG was added to 0.1 mM to induce protein expression. Bacteria were grown for approximately two more hours in the presence of IPTG and then harvested by centrifugation at 8,000 *rpm* for 10 minutes. Protein extract was made by resuspending a 200 mL culture in 10 mL of ice cold Buffer 1 (100 mM KCl, 50 mM HEPES, pH 7.5, 10% glycerol, 5 mM MgCl_2_, 1 mM DTT and protease inhibitors). The cells were lysed on ice by sonication and were visually monitored with a compound microscope to assess the efficiency of sonication. Lysates were cleared by centrifugation and the supernatant was retained. Fusion proteins were purified by binding to ~500 μL of a 50% slurry of glutathione Sepharose 4B that had been washed and equilibrated in Buffer 1. The protein was mixed with the Sepharose resin for 2 hour at 4°C and then loaded into a gravity flow column. The protein bound to the Sepharose was washed with 5 column volumes of Buffer 1. For the SELEX, the Sepharose beads with the bound purified protein was resuspended in 250 μL Buffer 2 (100 mM KCl, 50 mM HEPES, pH 7.5, 50% glycerol, 5 mM MgCl_2_, 1 mM DTT) and stored at 4°C. For gel shifts, the protein was eluted with glutathione by incubating the slurry for two hours in elution buffer (10 mM glutathione in 50 mM Tris, pH 8.0) and dialyzed into Buffer 1. Examination of protein by SDS-PAGE and Commassie staining showed a high degree of purity and proteins of the expected sizes.

The SELEX procedure was performed as previously described with some modification [24]. Here we used oligoR76: CAGGTCAGTTCAGCGGATCCTGTCGN_26_GAGGCGAATTCAGTGCAACTGCAGC, primer F GCTGCAGTTGCACTGAATTCGCCTC and primer R CAGGTCAGTTCAGCGGATCCTGTCG. For the first round of the SELEX procedure, a complementary strand of OligoR76 was generated by a single cycle of PCR using primer F, 100 ng of OligoR76 in a 20 μL PCR reaction to generate double stranded molecules (1 minute at 94°C, 3 minutes at 62°C, 9 minutes at 72°C). The first round of SELEX was performed with 5 μL of the primer extension oligoR76 DNA reaction, 20 μL of the GST-Fru/bead slurry and 100 μL of Buffer 3 (Buffer 1 with poly dI,dC 4 μg/μL and BSA 40 μg/mL) for 2 hours at 4°C. The protein/bead slurry was washed twice with 800 μL Buffer 1 and then was resuspended in 30 μL of high quality water, boiled for 2 minutes and the supernatant that included the bound DNA retained. For the next nine rounds of SELEX, 10 μL of the eluted DNA from each preceding round was used in a 100 μL PCR reaction for 20 cycles, using primers F and R. The DNA was loaded onto a 2% Nuseive gel and separated by electrophoresis. The DNA band at 75 base pairs was excised and purified using Qiaquick gel extraction columns (Qiagen). For the last rounds of the SELEX, 1 μL of the purified DNA from the previous round (~300 ng) was mixed with 20 μL of the protein/bead slurry in 100 μL Buffer 3. The bound DNA fragments were purified as described for round 1 of the SELEX. After the tenth round of the SELEX, the DNA fragments were cloned by ligation into Bluescript at the *EcoR*I and *BamH*I sites and sequenced by Sanger sequencing. For each Fru DBD, at least 20 independent clones were sequenced. A Gibbs sampling algorithm was used to find the consensus motif [45]. For Fru^MB^ a sequence of low complexity was identified from the first SELEX experiment and so a second full SELEX experiment was performed, each with 10 rounds of selection. Nearly the same consensus sequence was identified in both rounds, providing confidence in the result.

To determine if the motifs identified in the SELEX bind specifically to the DNA binding domain used in the SELEX procedure, gel shift reactions were performed using annealed phosphorylated oligonucleotides that contain common flanking DNA sequence chosen to facilitate cloning and either the binding sequences identified in the SELEX (in bold below), or oligonucleotides of the same nucleotide content but randomized but in the same position as the identified binding site resides in the sequence (in italic and bold below). For Fru^MA^ the oligonucleotide sequences were 5′TCGACCTGCAG**AGTAAC**CTGCAGG and 5′TCGACCTGCAG**GTTACT**CTGCAGG. The Fru^MA^ randomized sequences were 5′TCGACCTGCAG***ATAGAC***CTGCAGG and 5′TCGACCTGCAG***GTCTAT***CTGCAGG. For Fru^MC^ the oligonucleotide sequences were 5′TCGACCTGCAG**TGTTACATCA**CTGCAGG and 5′TCGACCTGCAG**TGATGTAACA**CTGCAGG. The Fru^MC^ randomized sequences were 5′TCGACCTGCAG***GCATCTATAT***CTGCAGG and 5′TCGACCTGCAG***ATATAGATGC***CTGCAGG. Gel shift reactions were performed as previously described [46].

For Fru^MB^, two independent trials with ten rounds of selection for the Fru^MB^ binding motif identified very similar sequences. The consensus sequence for Fru^MB^ from the two independent trials is **GCCCTTT**. The GST-Fru^B^ protein bound DNA in the invariant region present in all the synthesized oligos (see above). To determine if the binding site identified for Fru^MB^ was correct, a modified assay was performed in test tubes. In this assay 5 μL of P^32^ labeled DNA from the first and tenth round of the SELEX procedure were mixed with 30 μL of the Fru^MB^ protein/bead slurry in 150 μL Buffer 3 for two hours at 4°C, washed three times in Buffer 1 and added to 5 mL of scintillation fluid. The percent of the input retained from the first and tenth SELEX round was quantified using a scintillation counter. The P^32^ labeled DNA was generated by a standard PCR reaction, using Primer F and R, 1 μL of DNA from the SELEX round, and included dATP-P^32^gamma. Only 0.8% of the labeled input DNA from the first round of SELEX was retained on the Fru^MB^ protein/bead slurry, whereas 3.6% was retained from the tenth round of SELEX, demonstrating that the SELEX enriched for a sequence bound by Fru^MB^.

### Illumina read mapping

We used a sequential mapping pipeline that mapped approximately 95% of all reads to the Drosophila genome. Barcode, primer, and adapter sequences were trimmed. Reads were aligned to the *D. melanogaster* genome FB5.30 (FlyBase v5.30) using Bowtie (--tryhard, --best, --strata, -m1*)*[47], unaligned reads were 3′ end quality trimmed and homopolymers (5+) were removed. Quality trimmed reads were mapped as above. Unaligned reads at this step were aligned to junctions estimated by Tophat [48]. Any remaining unaligned reads were mapped allowing for gaps to FB5.30 using LAST [49]. Reads were visualized as wiggle tracks on FB5.30 genome using a custom R script [25].

Within a gene, exons from different isoforms may overlap due to alternative start and end positions (Additional file 10: Figure S4). Exons from different genes may also overlap. Overlapping exons, regardless of strand, were combined into the maximum exonic region see [25]. If there was a single exon in the region it was labeled as S####_SI and if there were multiple overlapping exons, they were combined and labeled as F####_SI (Additional file 10: Figure S4). Exonic regions can be further classified as constitutive (a single exon present in all isoforms), common (exonic region present in all isoforms), and alternative (not present in all isoforms) (Additional file 10: Figure S4). Expression was quantified for each exon in FB5.30 using a perl script. In regions where exons overlap, exons were combined. Of the 60,291 exonic regions 53,459 did not overlap with any other exon. Most overlaps are due to alternate start or end positions (4,503). About 30% (2,329) are due to exonic regions from different genes, these regions were not considered further. We considered an exonic region as detected if at least one read mapped to that region. Exonic regions with no reads mapping for any observed samples were not considered further (1,550 regions). For each exonic region, Reads Per Kilobase per Million mapped reads RPKM [50] was calculated and the natural log taken. If no variation was observed in one condition, or if it was not detected at least once in each treatment group no statistical analysis is possible. These 11,104 regions and the remaining 45,339 exonic regions that were analyzed for quantitative differences in expression are reported in Additional file 11: Table S7.

### Differential expression

A linear model was fit for each exonic region separately and models were examined for conformation to assumptions. All comparisons were performed as contrasts in a single model and a single FDR correction was performed for all contrast simultaneously [51]. Results were partitioned into induced or repressed based upon the direction of the observed difference. To declare that an exon was significantly differentially expressed, we required that exons be both (1) statistically significantly different [False Discovery Rate (FDR) p-value < 0.20] and (2) have a ≥2 fold change in expression level (Additional file 1: Table S1 and Additional file 3: Table S2). We also provide the full lists of genes that have exons that are significantly differentially expressed (Additional file 12: Tables S8 and Additional file 13: Table S9). To reduce the chances of identifying exons only due to background differences in strain, we required that the Fru^M^ be different from both wild type backgrounds in order to be declared differentially expressed. For the *fru* null comparisons, we required that both *fru* allele combinations were each statistically different from CS and Berlin (four statistical comparisons).

### Enrichments and motif analysis

Enrichments for chromosomal locations were tested by constructing contingency tables and conducting a Fisher’s exact test [52]. Position weight matrices (PWMs) generated by SELEX enrichment, were used to identify the locations of Fru^MA^, Fru^MB^, or Fru^MC^ binding sites. MAST [53] was used to identify putative binding sites in a region that included the gene of interest and 2 kb upstream of the transcription start site, 2 kb downstream of the 3′ UTR and throughout the entire genic region and did not attempt to normalize the counts. Enrichment of Fru binding sites in genes that were significantly induced or repressed was tested using a Fisher’s exact test [52]. Chromosomal enrichment for genes identified as differentially expressed was tested using a Fisher’s exact test for each chromosomal arm.

Gene Ontology (GO) enrichment analysis was performed using Gene Ontology enrichment analysis and visualization tool (GOrilla) [29]. Target list containing FBgns from genes either induced or repressed in the Fru^M^ over-expression or Fru^M^ loss-of-function were supplied against a background list containing all 14903 FBgns to obtain significantly enriched (p value < 10^-3^) GO terms for biological processes, cellular components, and molecular functions. Enriched protein domain analysis was implemented with the Holm-Bonferroni correction in the Flymine portal [28].

## Abbreviations

Dsx: Doublesex; fru: fruitless; GO: Gene Ontology; RNA-seq: RNA-sequencing; oligo: Oligonucleotide; DBD: DNA binding domain; CNS: Central nervous system.

## Competing interest

The authors declare that they have no competing interests.

## Authors’ contributions

JED, MNA performed experiments. JED, JF, SK, LMM and MNA contributed to data and statistical analyses. JED, JF, SK, BB, LMM and MNA contributed to writing the manuscript. All authors read and approved the final manuscript.

## Supplementary Material

Additional file 1: Table S1 Genes induced and repressed by Fru^M^ isoforms in males (gene lists are based on the statistical tests and the ≥ 2-fold difference criterion). Click here for file

Additional file 2: Figure S1 Venn diagrams of comparisons. Click here for file

Additional file 3: Table S2 Genes induced and repressed by Fru^M^ isoforms in females. (gene lists are based on the statistical tests and the ≥ 2-fold difference criterion). Click here for file

Additional file 4: Table S3 Genomic locations for Fru^M^ DNA binding motifs. Click here for file

Additional file 5: Table S4 Gene Ontology analyses. Click here for file

Additional file 6: Table S5 Genes induced and repressed by Fru^M^, as determined by examination of *fru P1* mutants and wild type males. All data described is included. Click here for file

Additional file 7: Table S6 Statistical analysis of chromosomal distribution of genes regulated by Fru^M^. Click here for file

Additional file 8: Figure S2 Fru^M^ is localized in the *fru P1*-expression pattern in flies over-expressing Fru^MA,B^ or ^C^. Click here for file

Additional file 9: Figure S3 Courtship analyses. Click here for file

Additional file 10: Figure S4 Schematic of how exon IDs are determined. Click here for file

Additional file 11: Table S7 Exon region ID information and all associated data from this study. Click here for file

Additional file 12: Table S8 Genes induced and repressed by Fru^M^ isoforms in males (gene lists are based on the statistical tests). Click here for file

Additional file 13: Table S9 Genes induced and repressed by Fru^M^ isoforms in females (gene lists are based on the statistical tests). Click here for file
