# Supplementary material for: Male-specific Fruitless isoforms have different regulatory roles conferred by distinct zinc finger DNA binding domains
Source: BMC Genomics. 2013 Sep 27;14:659. doi: 10.1186/1471-2164-14-659 (PMC3852243; doi:10.1186/1471-2164-14-659)
Supplement: Additional file 2: Figure S1 — Venn diagrams of comparisons. [file 1471-2164-14-659-S2.pdf]

Additional Figure 1

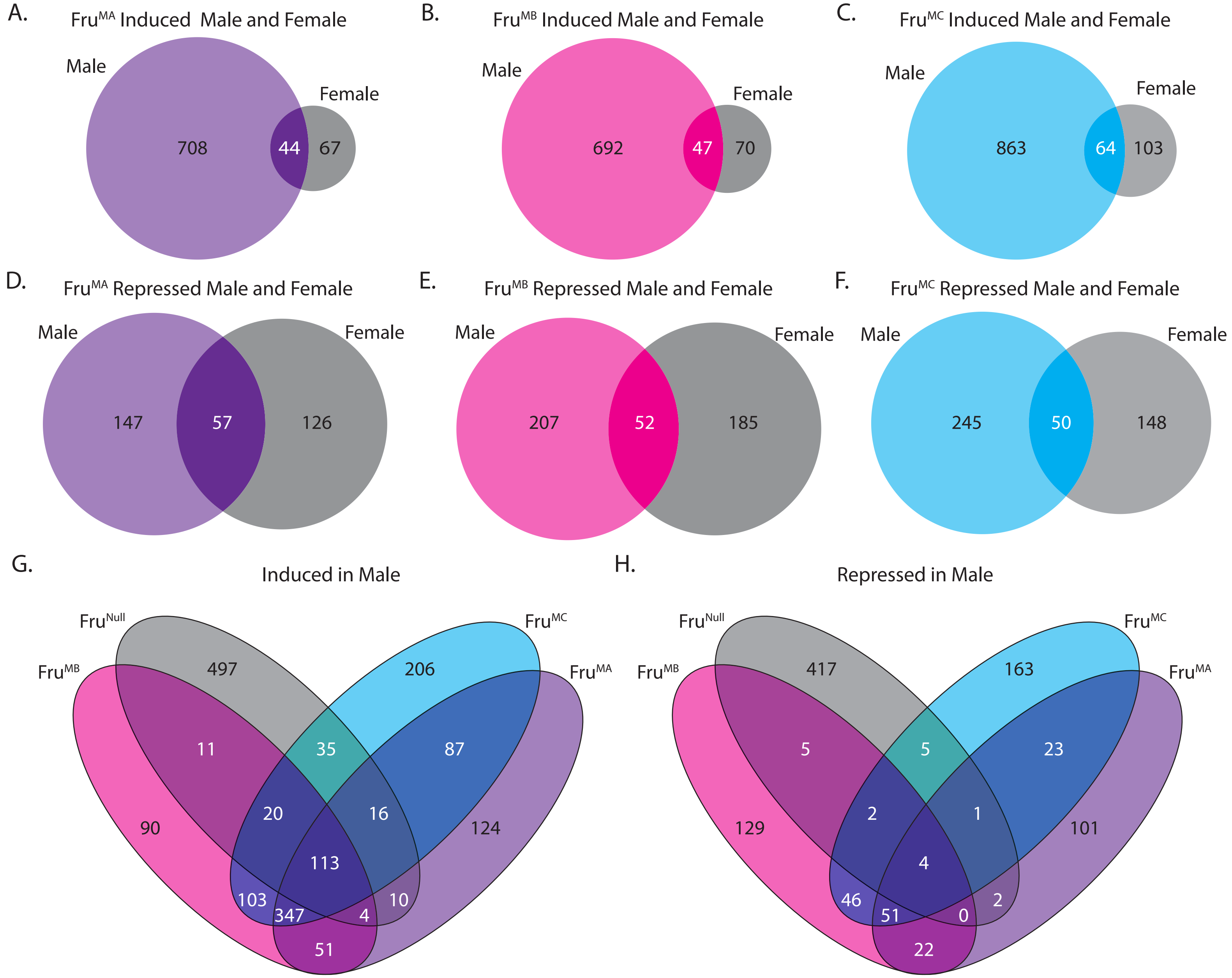

Additional Figure 1: Venn diagrams of comparisons from various sets of genes.

(A-H) Venn diagrams displaying number of distinct and mutual genes between different sets of differentially expressed genes.

(A-F) Numbers of genes induced or repressed in males and females by over-expression of FruMA, FruMB or FruMC in fru P1-expressing neurons of the head.

(G-H) Numbers of genes induced or repressed by FruM as determined by FruM loss of function analysis, and induced or repressed by over-expression of FruMA, FruMB, or FruMC isoforms.
