# Supplementary material for: Male-specific Fruitless isoforms have different regulatory roles conferred by distinct zinc finger DNA binding domains
Source: BMC Genomics. 2013 Sep 27;14:659. doi: 10.1186/1471-2164-14-659 (PMC3852243; doi:10.1186/1471-2164-14-659)
Supplement: Additional file 8: Figure S2 — FruM is localized in the fru P1-expression pattern in flies over-expressing FruMA,B or C. [file 1471-2164-14-659-S8.pdf]

Additional Figure 2

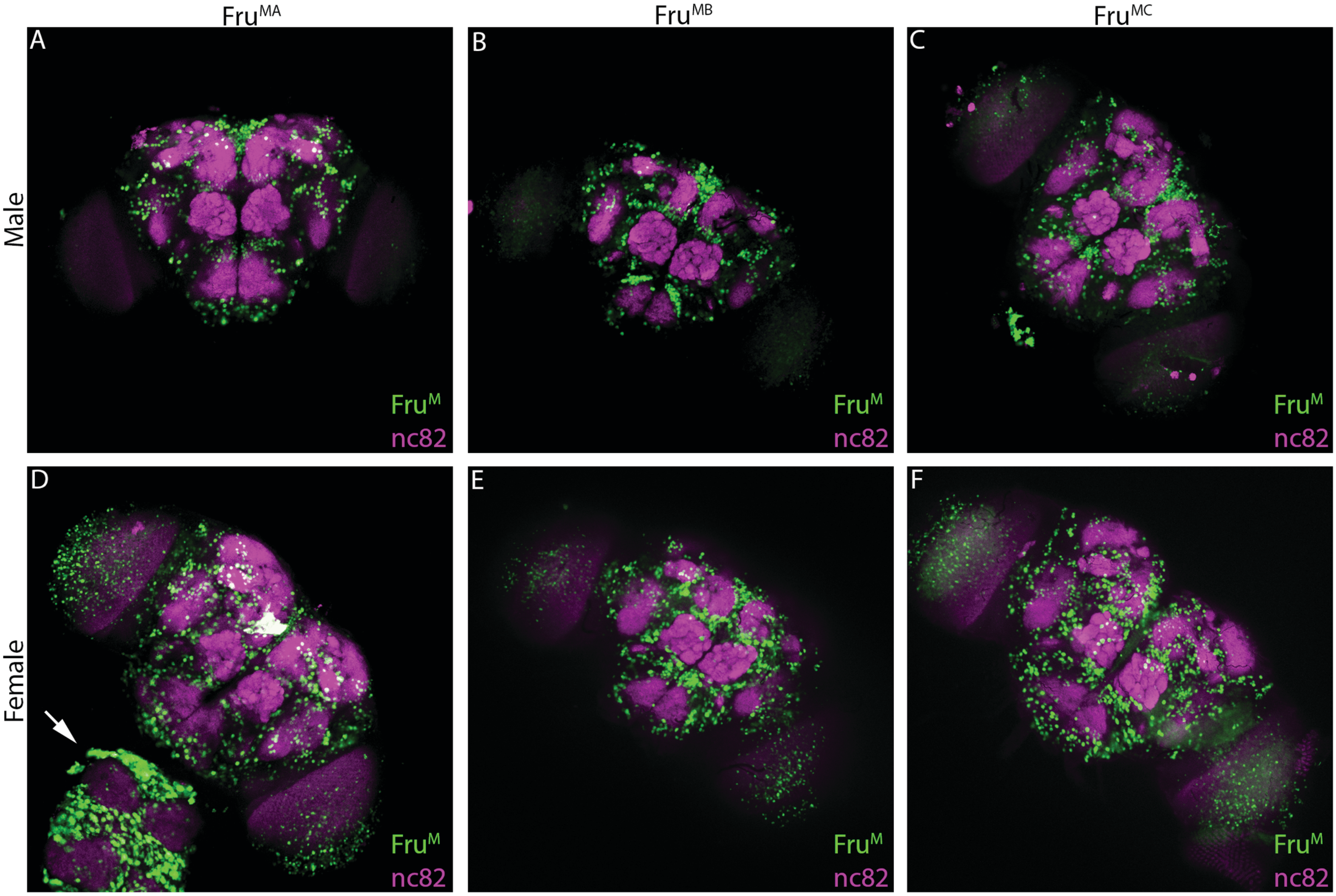

Additional Figure 2: Fru^{M} is localized in the *fru P1*-expression pattern in flies over-expressing $Fru^{MA,B\ or\ C}$.
(A-F) Immunofluorescence of Fru^{M} antibody (green) and the pan-neuronal nc82 antibody (purple) of brains from 0-24 hour adults male (A-C) and female (D-F) animals over-expressing Fru^{MA} (A and D), Fru^{MB} (B and E) or Fru^{MC} (C and F) in *fru P1*-expressing neurons.
[genotypes are: *y w/(w or Y); P(w+mC, UAS-Gal4)/P(w+mC, UAS-*$Fru^{MA,\ B\ or\ C}$); *fru P1-Gal4/+*].
Images were captured using 20x objective and confocal projections of the anterior z-slices of the brains are shown.
Arrow indicates a portion ventral nerve cord.
