# Supplementary material for: Male-specific Fruitless isoforms have different regulatory roles conferred by distinct zinc finger DNA binding domains
Source: BMC Genomics. 2013 Sep 27;14:659. doi: 10.1186/1471-2164-14-659 (PMC3852243; doi:10.1186/1471-2164-14-659)
Supplement: Additional file 9: Figure S3 — Courtship analyses. [file 1471-2164-14-659-S9.pdf]

Additional Figure 3

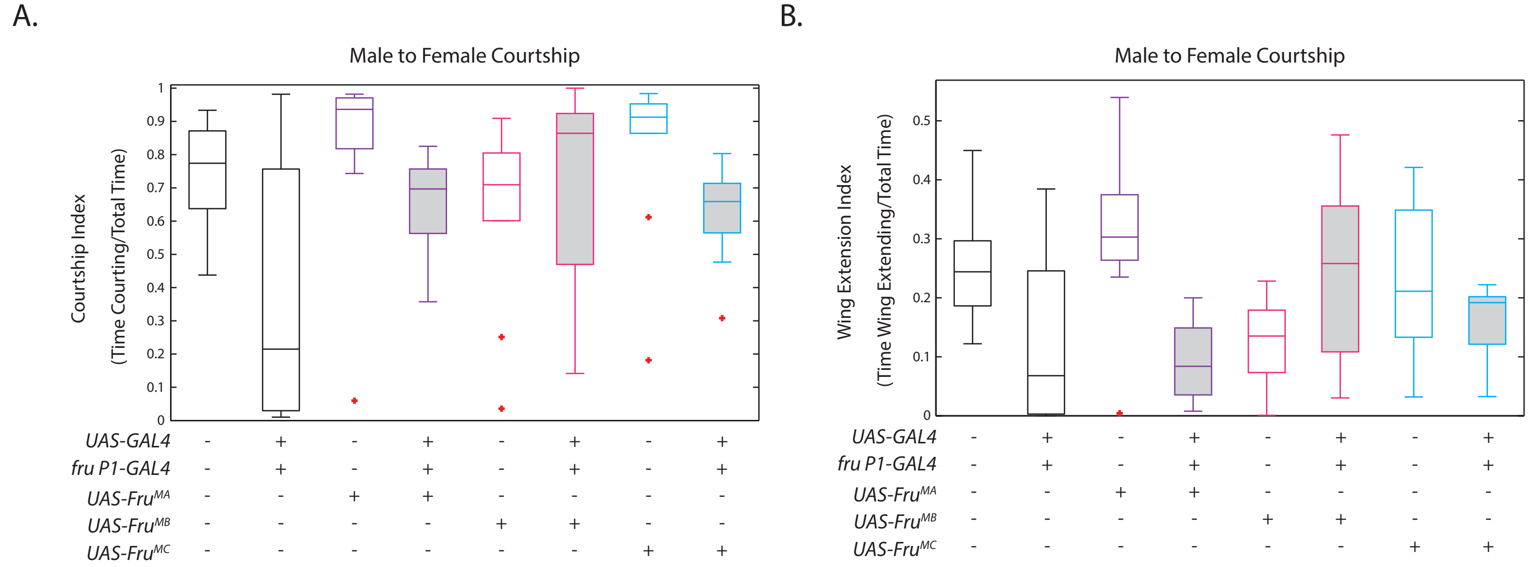

Additional Figure 3: Courtship analysis of males over-expressing Fru$^{MA,B\ or\ C}$.
Courtship index (A-B) and wing extension index (C-D) for male-female (A and C) and male-male (B and D) courtship behavior of corresponding experimental or control males (grey and white boxes, respectively). Males over-expressing Fru^{MA}, Fru^{MB}, or Fru^{MC}in *fru P1*-expressing neurons (grey filled purple, pink or cyan), single transgene Fru^{MA}, Fru^{MB}, or Fru^{MC}males (white filled purple, pink, or cyan), *UAS-GAL4/+; fru P1-GAL4/+* single transgene males, and Canton S males (no transgenes indicated) are indicated. Dashed grey box indicates statistically significance difference of experimental males from all three controls (Canton S, *UAS-GAL4/+;fru P1-GAL4/+*, and respective Fru$^{MA,B\ or\ C}$ single transgene males) at p-value < 0.05 (Wilcoxon rank sum test, two sided). Median is indicated by horizontal line in box, boxes cover 25th to 75th percentiles of the samples, whiskers represent data not considered an outlier, and outliers are defined as values greater than or less than 1.5 times the interquartile range (red cross).
