# Supplementary material for: Male-specific Fruitless isoforms have different regulatory roles conferred by distinct zinc finger DNA binding domains
Source: BMC Genomics. 2013 Sep 27;14:659. doi: 10.1186/1471-2164-14-659 (PMC3852243; doi:10.1186/1471-2164-14-659)
Supplement: Additional file 10: Figure S4 — Schematic of how exon IDs are determined. [file 1471-2164-14-659-S10.pdf]

Additional Figure 4

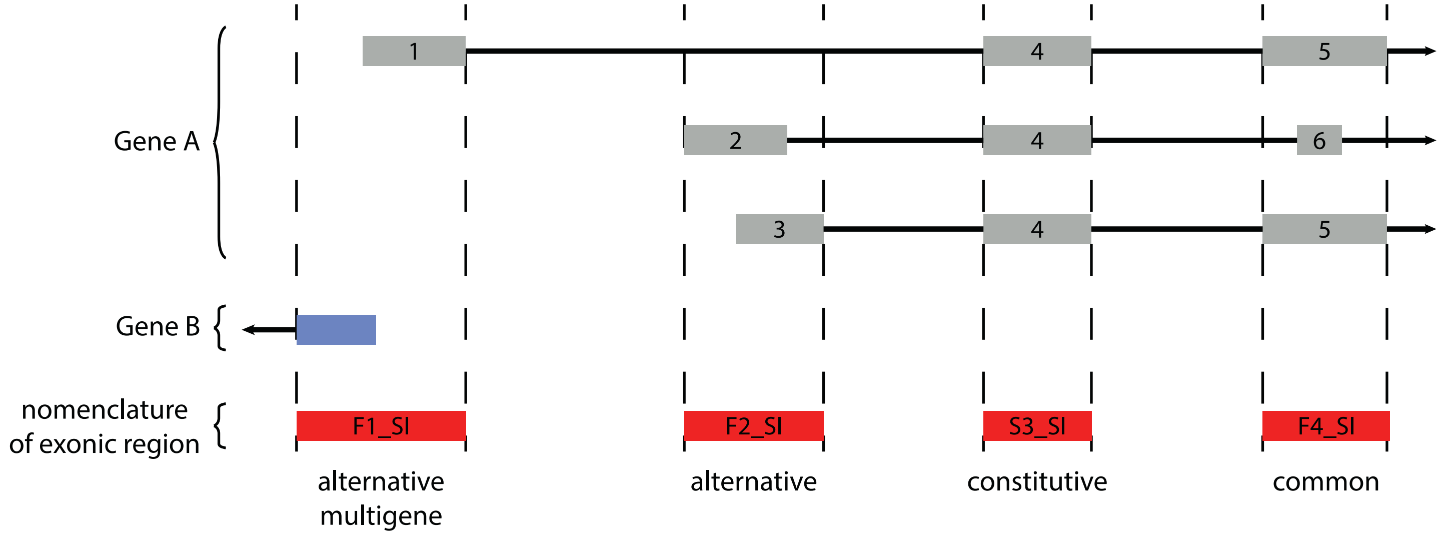

Additional Figure 4: Illustration of Exonic region design and nomenclature.
Exonic regions were classified as either belonging to a single exon, S#_SI, or multiple overlapping exons, F#_SI. Multiple overlapping exons consist of exons that were from different genes (alternative multigene), overlapping exons due to alternative start and end positions (alternative), or exonic regions present in all isoforms (common).
